# Multi-Objective Optimization of an Injection Molding Process for an Alvarez Freeform Lens Using an Integrated Optical System and Mold Flow Analyses

**DOI:** 10.3390/polym17182453

**Published:** 2025-09-10

**Authors:** Po-Yu Yen, Chao-Ming Lin, I-Hsiu Chang Chien

**Affiliations:** Department of Mechanical and Energy Engineering, National Chiayi University, Chia-Yi 600355, Taiwan; yenboyu@gmail.com (P.-Y.Y.); cjihsiu@gmail.com (I.-H.C.C.)

**Keywords:** Alvarez lens, mold flow analysis, Taguchi method, GRA, RMCO, spot diagram, distortion, MTF

## Abstract

This study optimizes the design and fabrication of an injection-molded Alvarez freeform lens using Moldex3D mold flow analysis and CODE V optical design simulations. The dual-software approach facilitates the transition between the manufacturing simulations and the optical design/verification process, thereby addressing the conversion issues between the two analysis modules. The optical quality of the designed lens is evaluated using spot diagram, distortion, and modulation transfer function (MTF) simulations. The Taguchi design methodology is first employed to identify the individual effects of the key injection molding parameters on the quality of the fabricated lens. The quality is then further improved by utilizing two multi-objective optimization methods, namely Gray Relational Analysis (GRA) and Robust Multi-Criteria Optimization (RMCO), to determine the optimal combination of the injection molding parameters. The results demonstrate that RMCO outperforms GRA, showing more substantial improvements in the optical quality of the lens. Overall, the proposed integrated method, incorporating Moldex3D, CODE V, Taguchi robust design, and RMCO analyses, provides an effective approach for optimizing the injection molding of Alvarez freeform lenses, thereby enhancing their quality. Future research could extend this methodology to other optical components and more complex optical systems.

## 1. Introduction

Injection molding, characterized by short cycle times and a net-shape manufacturing capability, is a cost-effective method for producing both optical prototypes and final products. The injection molding process encompasses several stages, ranging from material selection and the mechanical fabrication of the mold to the production of consumer-ready optical devices such as smartphone cameras, gaming equipment, audiovisual gear, and contact lenses [1,2,3].

**Figure 1:**
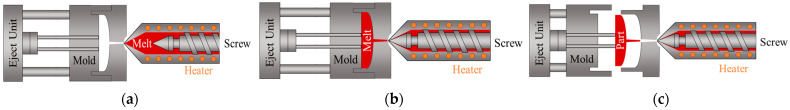
Main steps in injection molding process: (**a**) heating/melting, (**b**) filling/packing, and (**c**) ejection/cooling.

The choice of feedstock material depends on many factors, including the application requirements; the mechanical, thermal, and chemical properties; the aesthetic requirements; the cost; and the availability. Common injection molding materials include, but are not restricted to, acrylonitrile butadiene styrene (ABS), polypropylene, polyethylene, and polymethyl methacrylate (PMMA). The mold design phase considers the required functionality, dimensions, geometry (e.g., planar, curved, symmetrical, or asymmetrical), roughness specifications, and surface quality of the final product. The molding process begins with feeding plastic pellets into a hopper, where they are subsequently transferred to a plasticizing screw. As the screw rotates, the pellets are mixed and melted by external heating elements and conveyed to the runner and gate system. Under high pressure and controlled velocity and temperature conditions, the molten plastic is injected into the mold cavity, where it fills the mold and is then further compressed in a packing process. Once the molten resin has been compacted, the component is allowed to cool slightly in the mold. The ejector system then extends, causing the component to be ejected from the mold and to cool gradually to room temperature. After the component has been ejected, the mold closes again to commence the next production cycle [4,5]. The main steps in the molding process are illustrated in Figure 1. Injection molding offers the potential to produce parts with an exceptionally high surface finish and tight dimensional tolerances. However, to achieve this level of quality, the process parameters, such as the injection speed, mold temperature, cooling time, packing pressure, and melt temperature, must be carefully specified during the design stage and precisely controlled during production.

With its ability to mass produce plastic components of intricate shapes with high precision and reproducibility, injection molding is widely used in the optics field to fabricate lenses, light guides, prisms, diffusers, and many other optical components. Many studies have demonstrated the feasibility of injection molding for the high-volume production of Zernike freeform optical elements. Such surfaces find many applications, including wavefront aberration correction, ophthalmology, image analysis, and astronomy [6,7,8].

**Figure 2:**
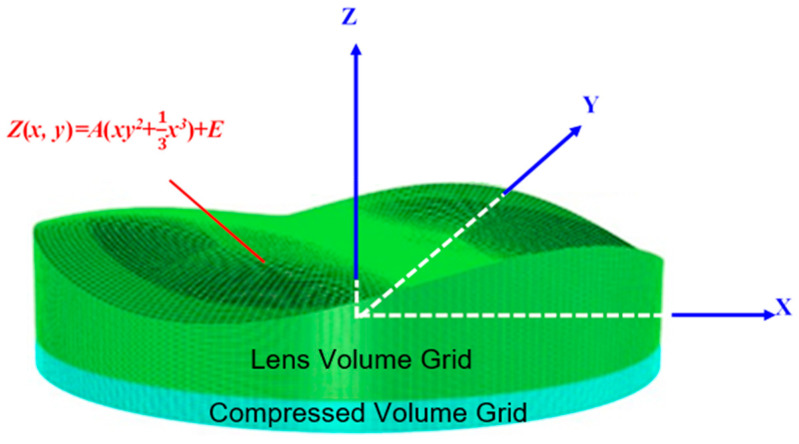
Surface profile of Alvarez lens.

One particularly interesting application of injection-molded freeform optics is the Alvarez lens, which is typically produced using polymethyl methacrylate (PMMA). The geometric design of Alvarez lens systems comprises two Alvarez lenses arranged in series. The resulting combination behaves as a spherical lens that utilizes lateral movements of the two elements to vary the optical power. Such functionality is achieved by creating optical thickness profiles with complementary phase plates, as illustrated in Figure 2 [9].

**Figure 3:**
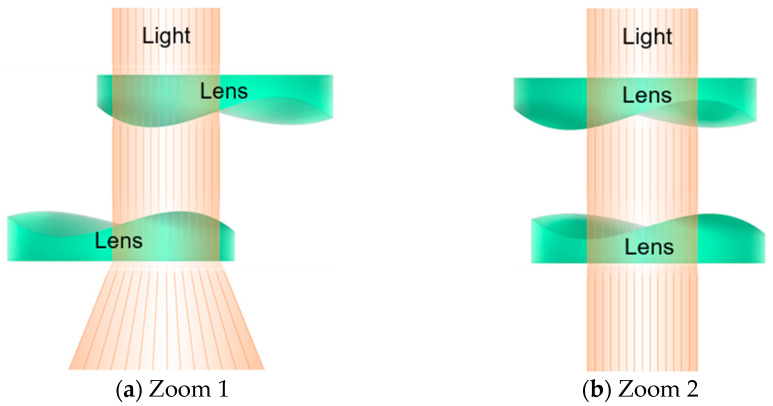
Optical configuration of Alvarez lens system in two operation modes (Green indicates: Lenses; Orange indicates: Light): (**a**) wide-angle (Zoom 1) and (**b**) telephoto (Zoom 2).

As shown in Figure 3, the optical elements in Alvarez lens systems display complementary cubic phase profiles. Consequently, even very small lateral displacements of the lenses relative to one another are sufficient to generate significant changes in the focus. In contrast, traditional zoom systems, which require at least two lenses, operate by changing the separation distance between the lenses to adjust the focal length and back focal distance. More complex zoom systems involve multiple lens groups that move independently while maintaining a fixed image plane. However, these lens systems typically rely on mechanical cam mechanisms and are prone to failure or jamming after prolonged use [10].

In Alvarez lens systems, the distance between the lenses remains fixed. Consequently, they excel in applications requiring compactness, reliability, and continuous zooming capabilities while avoiding the mechanical complexity and environmental limitations of conventional zoom lens systems. Generally, the elements in Alvarez lens systems are refractive. Using refractive optical elements avoids issues such as diffraction efficiency, chromatic aberration, and polarization sensitivity associated with liquid-crystal components.

Recent advancements have made possible the fabrication of adaptive focusing lenses based on liquid crystal and spatial light modulators, which offer non-mechanical zoom capabilities. These systems eliminate the need for multiple components and achieve variable focal lengths by altering the refractive index, thereby avoiding the requirement for mechanical movement. However, current technological constraints limit the achievable magnification range. Other challenges include power consumption, diffraction efficiency, chromatic aberration, and polarization effects [11,12]. As a result, there is a strong interest in the design and application of Alvarez lens systems.

**Figure 4:**
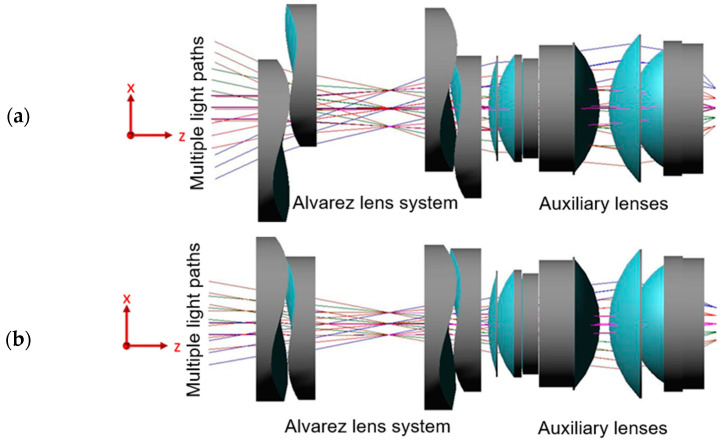
Multiple lens combinations used for imaging experiments in CODE V(2023.03) simulation software: (**a**) Zoom 1 and (**b**) Zoom 2.

**Figure 5:**
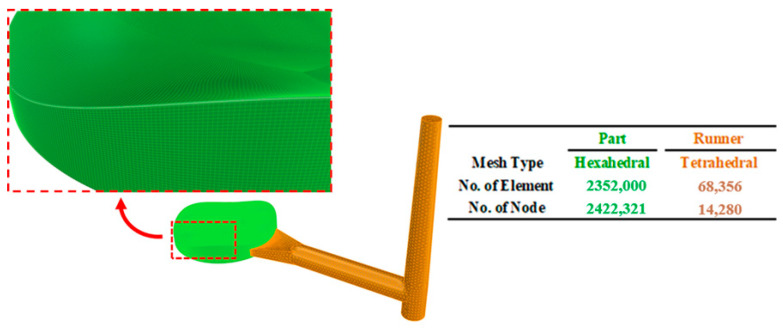
Grid and mesh for MFA simulation of gate and runner system for the injection molding of an Alvarez lens.

**Figure 6:**
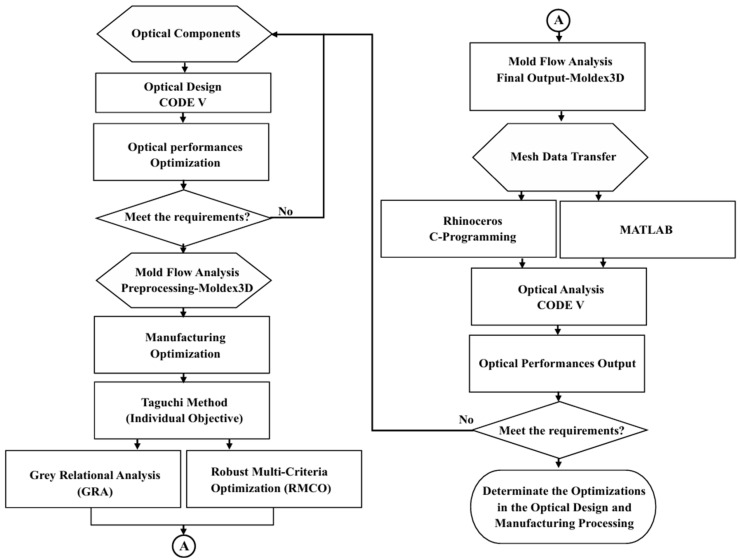
Flowchart of overall analysis framework.

In practice, the optical performance of Alvarez lenses depends on both the geometric design of the lens and the quality of the molded component. Consequently, an integrated consideration of the lens design and injection molding process is required. To meet this need, this study optimizes the design and fabrication of an injection-molded Alvarez freeform lens using CODE V (2023.03) optical design simulations (see the optical system combinations in Figure 4) and Moldex3D (2022.R4OR) mold flow analyses (see the grid and mesh for simulation of the gate and runner system in Figure 5). The optical quality of the designed lens is evaluated using the spot diagram, distortion, and modulation transfer function (MTF) methods. The optimization process commences by using the Taguchi methodology to identify the individual effects of the key injection molding parameters (e.g., the material type, mold temperature, filling time, and pressure) on the quality of the fabricated lens. Two multi-objective optimization methods, namely Gray Relational Analysis (GRA) and Robust Multi-Criteria Optimization (RMCO), are then separately employed to determine the optimal combination of the molding parameters. Finally, the optimization performance of the two methods is evaluated and compared [13]. The overall research framework is illustrated in Figure 6.

## 2. Mold Flow Analysis: Warpage, Stress, and Multiple Regression

As shown in Figure 6, the simulation process commenced by performing a preliminary mold flow analysis (MFA) of the initial lens design under default settings of the injection molding parameters. The resulting deformed geometry information and residual stress data at the discrete mesh coordinates were converted into continuous functions using multiple regression equations. Optical analyses were then conducted to evaluate the quality of the lens in terms of the spot diagram, distortion, and modulation transfer function (MTF) simulations. The geometry design of the lens was adjusted, if necessary, and the MFA and optical analysis tasks were repeated. The process continued iteratively in this way until the optical performance satisfied the specified quality requirements. The details of the integrated simulation procedure are described in the following subsections.

### 2.1. Mold Flow Analysis

Mold flow analysis (MFA) is a numerical simulation technique used to predict the flow of a polymer melt within a mold cavity. The numerical models used in MFA are founded on a continuum assumption, which treats the molten polymer as a continuous medium. In the present study, the three-dimensional transient flow fields during the polymer injection and mold filling stages were computed using the High-Performance Finite Volume Method (HPFVM). In the packing pressure phase, the Finite Element Method (FEM) and Finite Difference Method (FDM) were used to calculate the residual stress and temperature gradient in the in-plane and through-thickness directions, respectively.

Mold flow simulations rely on the construction of effective mesh structures consisting of interconnected nodes and edges. Optimal mesh generation ensures precise numerical analysis and improves the convergence. This study employed Rhinoceros 5.0 as the primary modeling platform for constructing geometric models of the Alvarez lens elements and Moldex3D-IM (2022.R4OR) as the main simulation module. When building the mold surface elements in Rhinoceros 5.0, it is essential that the coordinate dimensions of these elements adhere to the nominal CAD model of the Alverez lens. Furthermore, the primary analysis engine in Moldex3D (2022.R4OR) is based on a finite element model, which requires perfect nodal connectivity between adjacent elements to ensure accurate results. Thus, local mesh adjustments were performed as necessary to address geometric discrepancies or abrupt changes in order to enhance the quality of mesh processing and the reliability of the simulation outcomes [14,15,16,17,18,19].

### 2.2. Molding Materials and Geometry Mesh

The MFA simulations considered two types of PMMA from the CHI-MEI ACRYREX series as the lens material: CM 205 and CM 211. Both materials are known for their favorable light transmission properties and were selected from the internal material library of the Moldex3D (2022.R4OR) software (see Table 1). The primary research target of the MFA model was the first Alvarez lens element in Figure 4. The lens was modeled using the Moldex3D (2022.R4OR) mesh generator, with a 0.025 mm grid size for the component and a 0.2 mm grid size for the runner mesh. The runner included a fan-shaped gate and a cold-slug well. The lens was meshed using hexahedral elements, while the runner and gate were meshed using tetrahedral elements. The mesh details are shown in Figure 5. 

The deformed surface mesh output from the MFA simulations was fitted to a smooth surface to ensure that the subsequent optical ray-tracing simulations were not impaired by non-smooth transitions at the mesh boundaries. A convergence analysis was conducted on the mesh size used in the numerical analysis. It was found that as the value of the hardness parameter decreased, the differences between the node distances also decreased. The maximum and average displacements both converged at a hardness of 0.1. Using excessively low hardness may cause overfitting issues outside the sampled points. Consequently, the hardness parameter was set at 0.1 in the MFA simulations to characterize the deformation features introduced during manufacturing (see Table 2).

### 2.3. Warpage and Residual Stress

During the injection molding process, local differences in the part thickness, geometry, and material properties lead to non-uniform cooling rates. This results in uneven shrinkage effects that cause warpage and deformation of the final product. While the stress within the molded part reduces during the packing and compression phases within the mold, a certain amount of residual stress inevitably remains after it is ejected from the mold and cools to room temperature. Previous studies have shown that the magnitude of this residual stress is affected by many factors, including the gate design, viscous flow behavior, molding conditions, free shrinkage, stress-induced deformation, temperature gradient, and material characteristics. For plastic lenses, the residual stress impacts both the mechanical and optical performance of the lens [20,21]. Among the various factors influencing the intensity of the residual stress, the molding conditions are particularly dominant. Thus, the molding process parameters must be carefully selected and controlled to minimize or mitigate these effects, or at the very least, make them predictable so that they can be factored into the original lens design [22].

### 2.4. Stress and Refractive Index

The change in optical properties of a material in response to internal stress can be described using the stress–optical relationship. The change in the light polarization properties of the material is manifested by a birefringence effect, with the result that the residual stress can be evaluated using a stress viewer technique [23,24]. In the present MFA simulations, the computed residual stress data were used in conjunction with the stress–optical law to estimate the distribution of the refractive index variations in accordance with

(1a) $$n_{1}-n_{0}=c_{1}\sigma_{1}+c_{2}\left(\sigma_{2}+\sigma_{3}\right)$$

(1b) $$n_{2}-n_{0}=c_{1}\sigma_{2}+c_{2}\left(\sigma_{3}+\sigma_{1}\right)$$

(1c) $$n_{3}-n_{0}=c_{1}\sigma_{3}+c_{2}\left(\sigma_{1}+\sigma_{2}\right)$$

where *n*_1_, *n*_2_, and *n*_3_ are the refractive indices corresponding to the principal stress directions; n_0_ is the refractive index of the material (CM 205 or CM 211) in its unloaded state; σ_1_, σ_2_, and σ_3_ are the principal stresses; and c_1_ and c_2_ are the stress–optical coefficients.

### 2.5. Multiple Regression

As described at the beginning of Section 2, the deformed geometry information and residual stress data obtained from the MFA simulations at the discrete mesh coordinates were converted into continuous functions using multiple regression (MR) equations. Optical analyses were then conducted to evaluate the quality of the lens in terms of the spot diagram, distortion, and MTF. The details of the conversion process are described below.

The Finite Element Analysis (FEA) results from the Moldex3D (2022.R4OR) simulations were output as two discretized data files: an (.OPTIC) file containing node and refractive index information and a (.CDB) file containing node and coordinate information. These files, once integrated, yielded discrete refractive index data at specific node coordinates. However, discrete geometric data and refractive index distributions are unsuitable for optical analysis simulations, which require continuous, smooth geometric features and globally defined refractive index functions. Accordingly, MR calculations were employed to convert the discrete geometric and refractive index data into functional representations. Using C programming (CODE V-2023.03) and nmake.exe (Windows Prompt-Visual Studio), the relevant data were transformed into a CODE V(2023.03)-readable (.DLL) file. The necessary files for CODE V(2023.03) optical analysis, including the CAD files, DLL files, and coefficient files derived from the MR process, were then imported into CODE V(2023.03) to evaluate the quality of the molded lens in terms of the three quality measures indicated above.

In general, MR equations can be expressed as y = θ_0_ + x_1_β_1_ + x_2_β_2_ + x_3_β_3_ + …, where y is the dependent variable, x_i_ are the independent variables, and β_i_ are coefficients. Typically, the parameter matrix β is obtained through matrix operations such as pseudo-inverse regression (PR). The MR fitting process performed in this study was implemented using Ahmet Cecen’s MultiPolyRegress function in MATLAB (R2023b) Central [25,26,27]. Briefly, the refractive index values at the coordinate points were computed using the extracted Moldex3D (2022.R4OR) FEA data and optical analysis files and were then converted into MR equations using the MultiPolyRegress function. The resulting polynomial equations described the refractive index field as a function of n(x, y, z).

However, CODE V(2023.03) does not natively support 3D gradient refractive index polynomials. Thus, dynamic link libraries (DLLs) were employed to implement the polynomial functions and their partial derivatives in the three spatial directions. The coefficients derived from the regression analysis were reformatted to serve as inputs for the custom DLLs. Following the user manual, the C code defining the refractive index function n(x, y, z) was compiled using Visual Studio’s command-line tool (NMAKE.EXE) to produce a DLL file. This DLL then allowed CODE V(2023.03) to simulate the refractive index field derived from the MATLAB (R2023b) regression analysis.

## 3. Optical System Design and Processing Optimization Methods

### 3.1. Optical Analysis System

The Alvarez lens system analyzed in this study consisted of two pairs of identical Alvarez lenses and a fixed-focus lens (see Figure 4). As shown in Figure 4a,b, the CODE V(2023.03) analyses considered two operation modes: a wide-angle mode (50° field of view, referred to here after as Zoom 1) and a telephoto mode (20° field of view, referred to as Zoom 2), with the effective focal lengths of the two modes differing by a factor of two. The detailed optical system specifications of the two modes are listed in Table 3. Collectively, the two operation modes provided nine fields of view, as listed in Table 4, with a wavelength of 590 nm in each case.

### 3.2. Taguchi Method

The Taguchi method employs statistical sampling techniques to design experiments using orthogonal arrays, perform variance analysis on experimental outcomes, and achieve robust single-objective process optimization. By integrating these statistical principles, the Taguchi method aims to improve both the process quality and the process robustness. It has found widespread use across diverse industries for applications such as manufacturing and production, product design, and quality control.

In this study, the Taguchi method was employed to investigate the individual effects of the main injection molding parameters on the quality of the molded Alvarez lens. In general, the Taguchi method begins by establishing a set of *b* control factors (parameters), each exhibiting *a* levels. These experimental conditions are then configured as a set of *n* experimental trials in an orthogonal array L_n_(a^b^). Here, the term “orthogonality” refers to the fact that the orthogonal array (OA) ensures a balanced representation of all possible combinations between any two factors (columns). For each run in the OA, the quality of the experimental outcome is evaluated using a carefully chosen signal-to-noise Ratio (SNR), where the form of this ratio (larger is better, smaller is better, or nominal is best) depends on the particular application under consideration [22,28,29,30,31,32,33]. After evaluating the SNR for each experimental trial, analysis of variance (ANOVA) is employed to analyze the differences among multiple groups and determine the influence of each factor on the quality metrics. The ANOVA results thus enable the identification of the optimal factor levels for the particular process considered.

Using Taguchi OAs to design experiments and then performing variance analysis allows for the calculation of several critical research metrics, such as the effect of factor *P* shifting from level *i* to level *i* + 1, denoted as $E_{P}^{i\to (i\;+1)}$, and the sum of squares SS_P_ for factor *P*. These calculations are given by Equations (2) and (3), respectively, as

(2) $$E_{P}^{i\to (i+1)}={{\bar{\eta }}_{Taguchi}}_{P(i+1)}-{{\bar{\eta }}_{Taguchi}}_{Pi}$$

(3) $$SS_{P}=\frac{n\times r}{L_{P}}\sum_{k=1}^{L_{P}}{\left({\bar{y}}_{Pk}-\bar{y}\right)}^{2}$$

where ${{\bar{\eta }}_{Taguchi}}_{Pi}$ is the average effect of factor *P* at level *i, n* is the number of experimental runs in the OA, *r* is the number of samples per run, *L_P_* is the number of levels for factor *P*, ${\bar{y}}_{Pk}$ is the mean quality characteristic at level *k* of factor *P*, and $\bar{y}$ is the overall mean quality characteristic.

The purpose of calculating the level effects and the sum of squares (*SS*) is twofold: first, to determine the optimal response level for each factor and second, to conduct ANOVA tests on the confidence level and contribution of each factor. In the Taguchi method, the contribution of factor *P* is defined as $Cont.=\frac{SS_{P}}{SS_{Total}}$, where $SS_{Total}$ is the total sum of squares. The F-test is used to determine the relative importance of the factors. In particular, the F-test is employed to compare the variance of the sample mean estimates ($mS_{z}^{2}$) with the population variance ($S_{y}^{2}$), as expressed in Equation (4).

(4) $$F=\frac{mS_{z}^{2}}{S_{y}^{2}}$$

where the numerator,$\;mS_{z}^{2}$, represents the variance resulting from factor changes, while the denominator,$\;S_{y}^{2}$, reflects the variance after eliminating all the factor influences.

A larger F-ratio indicates a smaller probability that the sample means belong to the same population, suggesting that the factor has a significant influence. Conversely, an F-ratio closer to 1 indicates that the factor exerts no meaningful impact on the process.

### 3.3. Gray Relational Analysis

Gray Relational Analysis (GRA) [34,35] is a modeling approach designed to handle systems that are incomplete and uncertain. It enables the analysis of the relative correlations between variables and objectives by converting experimental data into gray sequences, computing gray relational grades, and evaluating the relevant importance of different decision factors. In many data analyses, missing or unevenly distributed discrete data are common and often unavoidable. Most decisions are made based on such discrete points; for example, the Taguchi method uses OAs with minimal experiments to represent full-factorial experimental runs. This approach, although efficient, can lead to uncertainties in the relationships between variables, particularly when dealing with multiple objectives. Moreover, the Taguchi method is a single-objective optimization method. That is, it provides insights only into the individual effects of the various processing parameters on the quality outcome rather than their collective effects. Nonetheless, by combining GRA with the Taguchi method, the SNR results can be used as interpretive indicators, and gray relational grades can be generated using Deng’s gray model [36,37,38]. This process ultimately enables the formulation of a multi-objective optimization problem in which the aim is to determine the optimal combination of process factor level settings that maximizes the quality of the experimental outcomes.

Gray Relational Analysis employs a series of steps to simultaneously account for data from multiple objectives. The process begins with normalization in order to make all the objective values comparable. Gray relational coefficients are then generated using Deng’s gray relational model. By default, all the objectives are assumed to be equally important. Consequently, the gray relational grade is obtained simply by averaging the gray relational coefficients. The resulting gray relational grade sequence can then be used to calculate the factor responses following the Taguchi method. This step determines how the gray relational grades respond to each factor, allowing for the selection of optimal levels for each factor. Ultimately, this approach yields a gray relational optimized process parameter configuration. The original response sequence is normalized as follows:

(5) $${x_{i}}^{*}=\frac{{x_{i}}^{\left(0\right)}\left(k\right)-\min{x_{i}}^{\left(0\right)}(k)}{\max{x_{i}}^{\left(0\right)}(k)-\min{x_{i}}^{\left(0\right)}(k)}$$

where ${x_{i}}^{*}$ is the normalized value from the data, $\min{x_{i}}^{\left(0\right)}(k)$ is the smallest value of ${x_{i}}^{\left(0\right)}\left(k\right)$; and $\max{x_{i}}^{\left(0\right)}(k)$ is the largest value of ${x_{i}}^{\left(0\right)}\left(k\right)$.

In GRA theory, a gray relational coefficient equal to unity implies that the two sequences are completely related. The equations are given as follows:

(6) $${∆}_{ij}\left(k\right)=\left\|x_{i}\left(k\right)-x_{j}\left(k\right)\right\|$$

(7) $$\gamma \left(x_{i}\left(k\right),x_{j}\left(k\right)\right)=\frac{∆min+\delta ∆max}{{∆}_{ij}\left(k\right)+\delta ∆max}$$

where *i* = 1, 2, 3, …, *m*; *j* = 1, 2, 3…, *m*; *k =* 1, 2, 3, …, *n*; ${∆}_{ij}\left(k\right)$ is the absolute value difference between $x_{i}\left(k\right)$ and $x_{j}\left(k\right)$; $x_{i}\left(k\right)$ is the reference series; $x_{j}\left(k\right)$ is a specific comparison series; $\gamma \left(x_{i}\left(k\right),\;{\;x}_{j}\left(k\right)\right)$ is the gray relational coefficient; Δ_min_ is the smallest value; Δ_max_ is the largest value; and δ is the distinguishing coefficient, where $\delta \in \left[0,\;1\right]$. (Note that the distinguishing coefficient is an index used to distinguish among the factors and was assigned a value of 0.5 by default in the present study.) Having calculated all the gray relational coefficients, the corresponding gray relational grades were obtained as follows:

(8) $$\Gamma \left(x_{i},x_{j}\right)=\frac{1}{c}\sum_{k=1}^{c}\gamma \left(x_{i}\left(k\right),x_{j}\left(k\right)\right)$$

where $\Gamma \left(x_{i},\;x_{j}\right)$ is the gray relational grade, and *c* is the number of sequences.

In summary, in this study, the GRA method was used to optimize the geometric design and manufacturing conditions of the considered Alvarez lens by effectively balancing and improving multiple performance objectives in the injection molding process.

### 3.4. Robust Multi-Criteria Optimization

The Robust Multi-Criteria Optimization (RMCO) method [39,40,41,42] is a multi-objective optimization approach that integrates Pareto optimality, Analysis of Variance (ANOVA), and iterative experimentation. Pareto optimality, originally a socio-economic concept, describes a state in which one individual’s condition improves without deteriorating that of others. In the context of multi-objective optimization, it ensures that at least one objective gains without compromising the others.

Prior to RMCO execution, the Taguchi OA results for each objective were analyzed using ANOVA to determine the contribution ratio, which reflects the importance of each factor toward a given objective. The basic rules for the RMCO process were then set as follows: Basic Rule 1: For factors significantly impacting multiple or all objectives, select the level that optimizes at least one of these objectives. Basic Rule 2: For factors significantly impacting only one objective, choose the level that optimizes that objective, disregarding its effects on other objectives. Basic Rule 3: For factors with no significant impact on any objective, set the level freely within the limits imposed by process constraints.

Based on these principles, RMCO proceeded with iterative experimentation and refined the parameter settings by selecting the best configuration from each iteration. The iteration process followed four key rules: Iteration Rule 1: If multiple objectives had differing optimal levels under Basic Rule 1, the ranges containing these optimal levels were incorporated into further iterative experimentation, and new level averages were reassigned within those ranges. Iteration Rule 2: For factors governed by Basic Rule 2, the selected level remained fixed throughout the entire iterative process. Iteration Rule 3: In cases where Basic Rule 3 applied, the best level determined via GRA replaced designer discretion to enhance the optimization process. Iteration Rule 4: If the impact of a factor on all the objectives was generally moderate, its original level configuration was retained without adjustment during iterative experimentation.

## 4. Optical Analysis: Spot Diagram, Distortion, and Modulation Transfer Function

In contrast to traditional interpretations of MFA simulation results, which generally focus on issues such as warpage deformation, optical path difference, birefringence, and refractive index variations, this study aimed to improve the transition between the MFA and optical simulation environments. By addressing this integration challenge, the optical software can more accurately interpret the mold flow results and implement more effective optimization methods. In the optical simulation process, the chosen evaluation criteria included spot diagram, distortion, and MTF simulations. These three criteria, combined with two zoom modes of the optical system (see Figure 2), resulted in a total of six objectives within the Taguchi experimental design. It was therefore crucial to clearly define the ideal function for each objective. The aim of the Taguchi experiments was to minimize variability in the evaluated metrics of the manufactured parts such that, under ideal conditions, the quality outcomes perfectly coincided with those of the optical design. Consequently, the outcomes from the optical design served as the reference ideal functions.

Given the problem considered in the present study, the SNR was formulated such that smaller differences between the experimental outcomes and the optical design results were preferred, as expressed in Equation (9), where *n* represents the number of samples, $\bar{y}$ represents the individual sample values, and $S_{n}$ is the standard deviation of the sample population.

(9) $$SNR=-10\times log\left(\frac{1}{n}\sum_{i=1}^{n}y_{i}\right)=-10\times log\left({\bar{y}}^{2}+S_{n}^{2}\right)$$

The following sections describe the evaluation metrics and the methods used to calculate their values in greater detail.

### 4.1. Spot Diagram

The spot diagram serves as a visual tool for evaluating the imaging quality of an optical system. By simulating the light paths, spot diagrams illustrate how light rays from different fields of view converge after passing through the optical system. In the spot diagram, each ray is represented as a point and is derived from uniformly distributed sampling points on the object or light source. The positions of these points on the imaging plane indicate the focusing quality and overall optical performance of the system.

**Figure 7:**
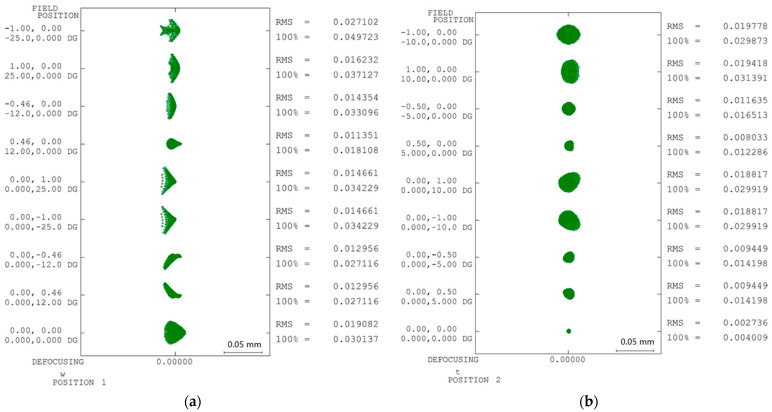
Spot diagrams of optical design: (**a**) Zoom 1 and (**b**) Zoom 2.

In this study, spot diagrams were constructed for nine fields of view under two zoom conditions (i.e., Zoom 1: wide angle; Zoom 2: telephoto). Figure 7a presents the spot diagrams for the nine fields of view in the wide-angle mode, while Figure 7b shows the spot diagrams for the telephoto mode. Ideally, when light passes through a perfect optical system, all the rays converge to a single focal point, resulting in a tightly clustered spot pattern. In other words, the ideal performance metric for a spot diagram is typically zero. However, because the manufacturing goal in the present study is to match the optical design rather than to optimize the design itself, the performance targets may vary depending on the magnitude of the results and their assigned weights. In some cases, these variations may even lead to optimization failures. Therefore, this study did not consider the ideal performance metric for spot diagrams to be zero. Instead, the ideal function was defined as the minimum root mean square (RMS) circular diameter achieved by the optical design in each field of view condition.

For any zoom mode, the quality characteristic $y_{i}$ under the spot diagram objective was determined as the difference in the minimum RMS diameter of the spot diagrams for that field of view before and after manufacturing variability, as expressed by Equation (10).

(10) $$y_{i}=\left|d_{i}-d_{design}\right|$$

where the variable $d_{i}$ represents the minimum RMS diameter of the spot diagram for a specific field of view, and *i* denotes the field number. Similarly, $d_{design}\;$is the minimum RMS diameter determined during the optical design phase. Given that there were nine fields of view in this study, *i* ranged from 1 to 9 in the spot diagram analysis.

### 4.2. Distortion

**Figure 8:**
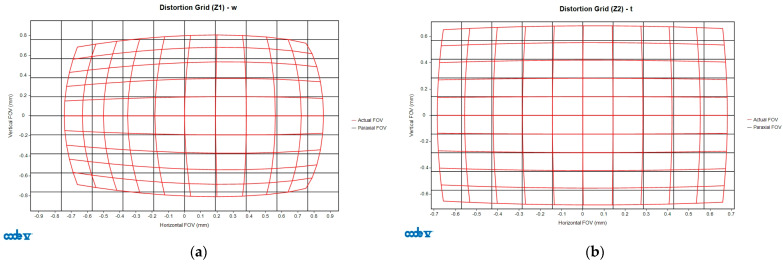
Deformation grid diagrams of optical design at FoV 0°: (**a**) Zoom 1 and (**b**) Zoom 2.

The deformation grid chart is a commonly used method to evaluate the distortion effects of an optical system. It measures optical deformation phenomena by using a grid of straight lines or squares to assess the distortion of an optical imaging system. For example, referring to Figure 8, the black gridlines represent the paraxial grid image, while the red gridlines show the imaging results. In this study, the distortion was evaluated exclusively at a field angle of 0°, and the node positions in the deformation grid for the optical design were compared to those affected by manufacturing variations. As for the interpretation of the spot diagram results, the quality characteristic $y_{i}$ was calculated as the distance between the XY plane coordinates of the design and manufactured grid points, respectively, as described in Equation (11).

(11) $$y_{i}=\bar{P_{design}P_{i}}=\sqrt{{\left(x_{i}-x_{design}\right)}^{2}+{\left(y_{i}-y_{design}\right)}^{2}}$$

where $P_{i}$($x_{i}$, $y_{i}$) represents the deformation grid nodes after manufacturing, and variable *i* denotes the grid point index. Similarly, $P_{design}$($x_{design}$, $y_{design}$) represents the deformation grid nodes during the design phase. Based on the automated grid setup in CODE V(2023.03), each side of the grid included 11 nodes, resulting in a total of 121 sampled data points. Thus, *i* ranged from 1 to 121 in the distortion analysis.

### 4.3. Modulation Transfer Function

**Figure 9:**
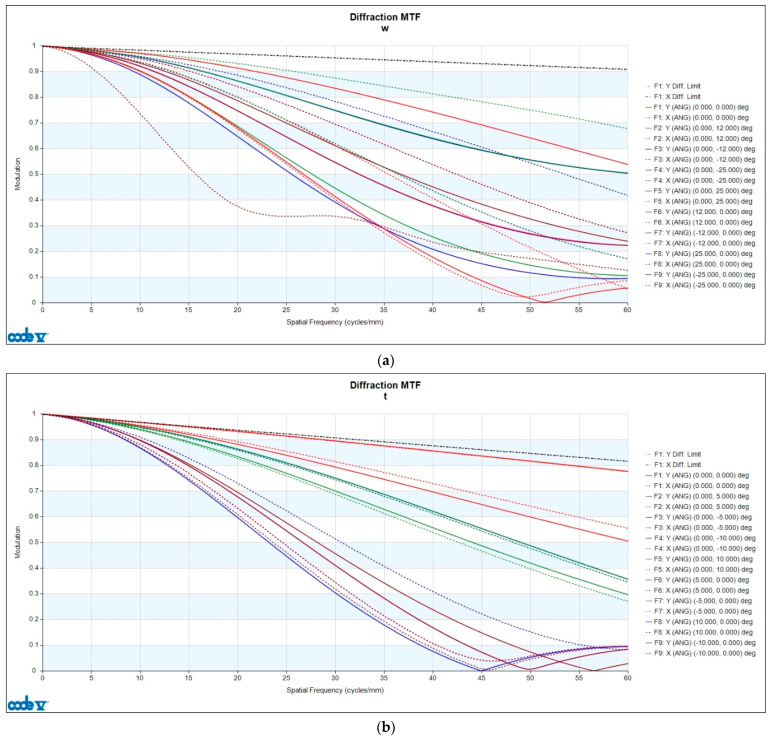
Diffraction MTF of optical design: (**a**) Zoom 1 and (**b**) Zoom 2.

The MTF describes the contrast transfer capability of an optical system at varying spatial frequencies and thus represents its ability to preserve image details. In the MTF curve, the horizontal axis denotes the spatial frequency, while the vertical axis indicates the modulation, reflecting contrast changes from 0 to 1. As shown in Figure 9, each field of view (Zoom 1 and Zoom 2) included both tangential and sagittal orientations, represented as X and Y in the right-hand legend.

There are two types of MTFs: geometric MTF and diffraction MTF. Geometric MTF describes the contrast degradation caused specifically by geometric aberrations in the optical system, excluding diffraction effects. Diffraction MTF is the product of geometric MTF and the diffraction limit, making it closer to real-world outcomes. Therefore, the analysis in this study focused on diffraction MTF. The analysis evaluated the MTF results up to 60 lp/mm (line pairs per millimeter) with an interval of 10 lp/mm. Since the result at 0 lp/mm approaches 1 infinitely, including it would diminish the observed differences. Therefore, the analysis was limited to the range of 10–60 lp/mm, where meaningful changes can occur. The quality characteristic *y_i_* was calculated as the modulation difference in the same field of view and direction, as expressed in Equation (12).

(12) $$y_{i}=\left|{Modulation}_{i}-{Modulation}_{design}\right|$$

where ${Modulation}_{i}$ represents the modulation value after manufacturing, while ${Modulation}_{design}$ refers to the modulation value from the optical design. With the data collected from nine fields of view, two orientations, and six spatial frequencies, a total of 108 data points are generated. Therefore, *i* ranged from 1 to 108 in the MTF analysis.

## 5. Results and Discussions

The aim of this study was to identify the combination of factors and levels for the injection molding process parameters that optimized the optical performance of the Alvarez lens system (see Figure 4). Notably, the design problem did not focus on a single optical component; instead, it involved a composite system capable of providing two distinct focusing modes. Consequently, achieving the desired outcomes for both modes (telephoto and wide-angle) and meeting the requirements of three optical quality metrics (spot diagram, distortion, and MTF) resulted in six optimization objectives. The Taguchi method is useful in this regard, as it allows for the efficient exploration of the factor space. However, it only provides single-objective optimization results, which are insufficient for the considered multi-objective problem. Therefore, the Gray Relational Analysis (GRA) and Robust Multi-Criteria Optimization (RMCO) approaches were also employed. These methods are particularly valuable in this context because of their capacity to handle and synthesize multifaceted optimization scenarios. GRA and RMCO enable the balancing of various performance effects and provide a comprehensive solution that simultaneously accounts for all six objectives, thus overcoming the limitations of the Taguchi method.

### 5.1. Determining Process Impact Factors

The initial step in the optimization process involved the identification and analysis of the critical process factors affecting the injection molding performance. Thus, a factor significance test was performed. Given the numerous factors influencing the quality of injection-molded components, an L_12_ orthogonal array (OA) was selected for the F-test experiment. The L_12_ OA, a specialized two-level orthogonal array, excludes interaction effects and is thus particularly suitable for factor screening via F-tests.

The level assignments for the control factors in the Taguchi design are presented in Table 5. As shown, the L_12_ OA incorporated ten factors: the feedstock material, mold temperature, melt temperature, filling time, multi-stage flow rate, packing time, packing pressure, V/P (velocity/pressure) switching ratio, ejection temperature, and cooling time. Two materials were considered: CM205 and CM211. For each material, the mold and melt temperature levels were interpolated between the upper and lower processing limits for the particular material. Levels at the extreme limits were denoted as Max or Min.

As shown in Table 5, column K in the OA was not assigned a factor and was reserved for “error.” This arrangement enabled the F-test in the Taguchi ANOVA stage to use the SNR (signal-to-noise ratio) variance results. In an OA without interactions, the final column serves as the error term for ANOVA, allowing for variance analysis of all considered process factors.

Table 6 shows the Taguchi experimental design and corresponding SNR results for each zoom mode and quality objective. Based on the factor screening results across all the objectives, six factors were identified as control factors for the main experiments: the material, mold temperature, filling time, filling rate (multi-stage), packing time, and packing pressure (see Table 7). To maximize the optimization benefits, the excluded factors were fixed at their optimal levels based on the GRA results. In particular, the melt temperature was set at Max, the ejection temperature at 90 °C, and the cooling time at 10 s. The V/P switch ratio exhibited only a minor effect across all the groups, and its two levels were split equally across the optimal results for each objective. Thus, given this balanced distribution and its minimal impact, a middle value of 97% was chosen for the V/P switching ratio.

### 5.2. Optical Performance Optimization Analysis

After the model is meshed, the influencing factors and levels are determined through the Taguchi method’s orthogonal array trial process. Each set of trials is then subjected to numerical mold flow analysis based on the specified parameter combinations. Each set of results contains discretized information, including the geometric coordinates and refractive index values of the mesh nodes. These discrete values must be converted into continuous data through fitting methods to facilitate subsequent optical analysis. The goal of this study was to identify the injection molding process parameters for an Alvarez lens that optimized three quality targets (spot diagram, distortion, and MTF) across two zoom conditions (a wide-angle mode and a telephoto mode). Table 8 presents the factor level assignments for the Taguchi experimental design used to satisfy this goal. In accordance with the Taguchi methodology, a variance analysis was performed on the experimental results. Two multi-objective optimization approaches (GRA and RMCO) were then employed to refine the optimal process parameters.

In general, multi-objective optimization requires careful consideration of the weighting configuration, that is, the objectives that are more critical for the product. Different weight proportions result in different optimal process configurations. As a result, the weights must be carefully assigned over the multiple objectives. However, in this study, all the objectives were considered equally important. Consequently, the objectives were assigned an equal weight. Altering the weight distribution would change the outcome. For both multi-objective optimization methods, the extent of process improvement for each combination of factor level settings was evaluated using the mean square deviation (MSD), which was evaluated as

(13) $$MSD=\frac{1}{n}\sum_{i=1}^{n}{\left(y_{i}-m\right)}^{2}=S_{n}^{2}+{\left(\bar{y}-m\right)}^{2}$$

where n  represents the sample size, $y_{i}$ denotes the individual data points, m is the target value for the data, and $\;S_{n}$ is the population standard deviation. The use of the MSD measure is motivated by the fact that all the objectives aim to closely match the optical design outcomes. Thus, if the data perfectly coincides with the optical design results, the optimization level can be expressed as 100%. If there is no improvement compared to the results for the baseline group, the improvement level is 0%. Conversely, if a group exhibits quality degradation, it can be represented as a negative gain, which indicates a loss.

As described above, six factors—the material, mold temperature, filling time, multi-stage filling rate, packing time, and packing pressure—were identified from the screening experiments as control factors for the main experiments. With the exception of the feedstock material, each control factor was assigned five level settings. Consequently, the experimental trials were configured in an L_25_(6^5^) orthogonal array. The material factor was arranged as a dummy level in the Taguchi experiments, meaning that a two-level factor was inserted into the five-level OA. That is, Levels 3 through 5 simply repeated the configurations of Levels 1 and 2 in the A-factor column of Table 8.

**Figure 10:**
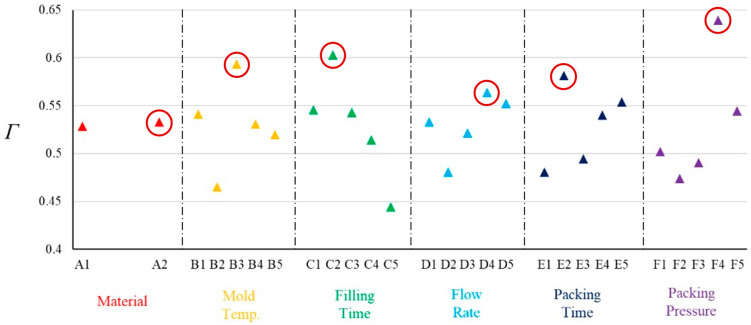
GRA response diagram. (The red circle indicates the largest correlation coefficient.)

Table 9 lists the SNR values for each experimental group and objective. The SNR values were used as inputs for the subsequent GRA optimization process. Table 10 presents the gray relational coefficient sequences and gray relational grade sequences for each objective. A factor response analysis was conducted on the gray relational grades. The results are shown in Figure 10, where the red circles indicate the optimal level selection for each factor based on the gray relational grade response analysis. Overall, the GRA results reveal that the optimal process configuration comprised the CM 205 material, a mold temperature of 50%, a fill time of 0.2 s, a four-stage filling rate, a packing time of 1.5 s, a packing pressure of 70%, and the fixed factor parameter settings.

### 5.3. Optical Performance Optimization Evaluation

The optimization outcomes for the Alvarez lens system were determined using three optimization methods: the standard process (parameters recommended by the molding machine), GRA, and RMCO. For each method, the spot diagram, distortion, and MTF performance were evaluated under both zoom conditions, and the results were compared with those of the optical design.

**Figure 11:**
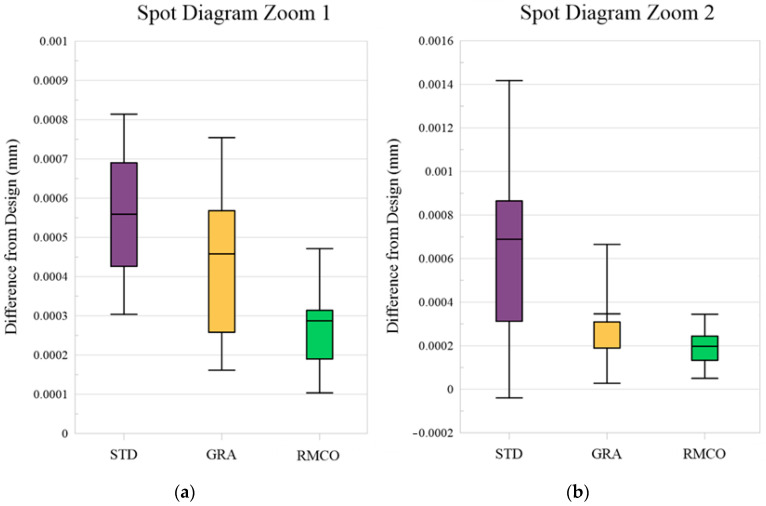
Box plots for spot diagram: (**a**) Zoom 1 and (**b**) Zoom 2.

**Figure 12:**
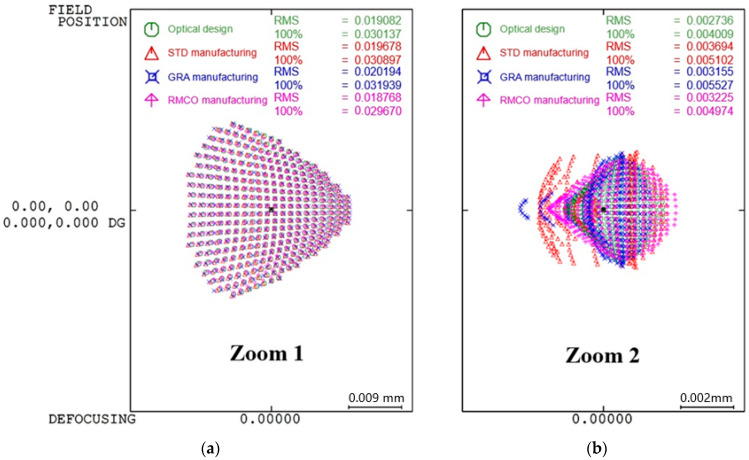
Spot diagrams: (**a**) Zoom 1 and (**b**) Zoom 2.

Spot Diagram Optimization: Figure 11 presents a box plot analysis indicating the spot diagram performance improvement obtained by each method compared with the optical design. The RMCO method resulted in the smallest deviation from the optical design. In particular, as shown in Figure 12, the RMCO method reduced the RMS value of the spot diagram to 0.018768 mm for Zoom 1 and 0.003225 mm for Zoom 2, demonstrating a notable enhancement compared to the optical design.

**Figure 13:**
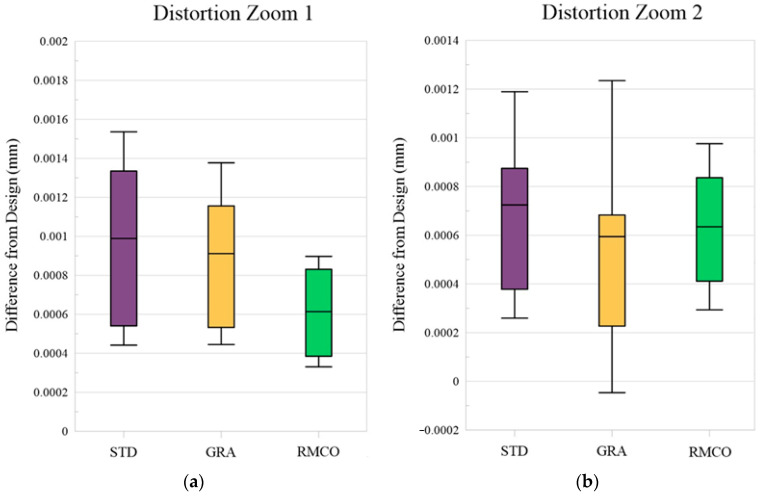
Box plots for distortion: (**a**) Zoom 1 and (**b**) Zoom 2.

**Figure 14:**
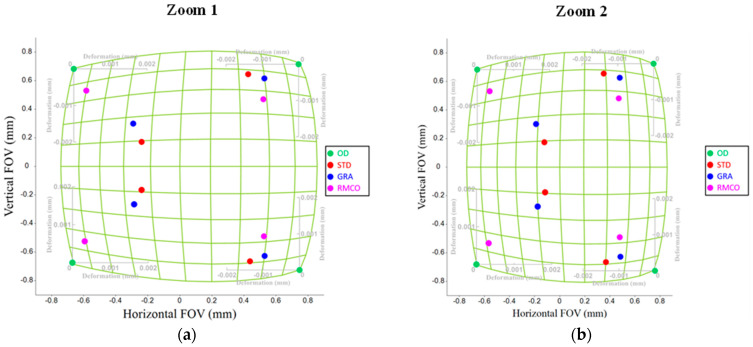
Distortion: (**a**) Zoom 1 and (**b**) Zoom 2.

Distortion Optimization: Figure 13 provides a box plot analysis of the distortion improvements obtained by the three methods. The RMCO method showed a clear improvement in the Zoom 1 (wide-angle) condition. However, its performance was slightly poorer than that of the GRA method for the Zoom 2 (telephoto) condition. Figure 14 shows the actual distortion maps with corner-point distributions. While all three optimization methods improved the overall distortion performance, they did not achieve noticeable improvements in the localized distortion.

**Figure 15:**
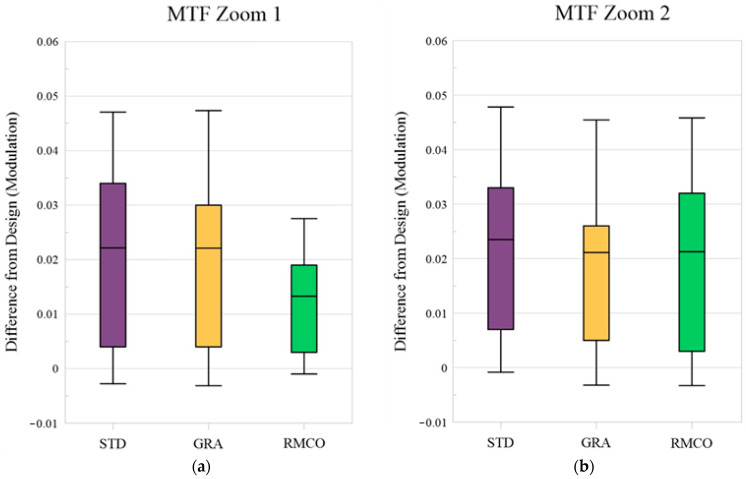
Box plots for modulation transfer function (MTF): (**a**) Zoom 1 and (**b**) Zoom 2.

**Figure 16:**
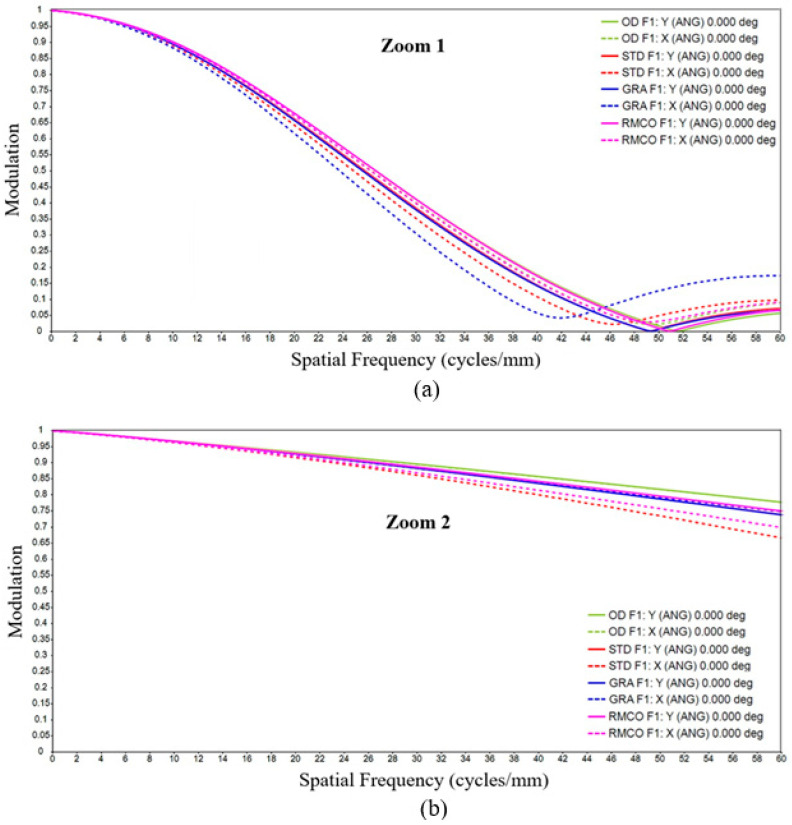
Modulation transfer function (MTF): (**a**) Zoom 1 and (**b**) Zoom 2.

MTF Optimization: As shown in the box plot in Figure 15, the RMCO method significantly improved the MTF performance in Zoom 1 but offered only a marginal improvement over GRA in Zoom 2. Figure 16 shows that all three optimization methods achieved a more pronounced MTF improvement in the Zoom 2 condition than in the Zoom 1 condition. Since Zoom 2 already exhibited strong MTF performance, further improvements were less evident, as corroborated by Figure 15.

RMCO Iterative Analysis **and** Best Parameter Selection: GRA and RMCO shared the same main trial setup, as shown in Table 9. Statistical significance and contribution levels were used to determine the factor importance. For the RMCO method, the factor levels were updated from GRA in accordance with the iteration rules described in Section 3.4. The updated configurations are listed in Table 11. New trials were then conducted using an L25 orthogonal array, with the results presented in Table 12. As shown, Group 10 achieved the highest mean gain, reducing the MSD by 55.182% and improving all the objectives. Specifically, the spot diagram improved by 69.583% (Zoom 1) and 93.849% (Zoom 2), the distortion improved by 64.194% (Zoom 1) and 29.881% (Zoom 2), and the MTF improved by 65.842% (Zoom 1) and 7.747% (Zoom 2). Based on Pareto optimality—without considering fairness issues—the best process parameters were determined from Group 10. Accordingly, the optimal process parameters were determined to be the CM 211 material, the maximum mold temperature, a 0.1 s filling time, a four-stage filling rate, a 1.5 s packing time, an 85% packing pressure, and the previously mentioned fixed factors, achieving a 55.182% average MSD reduction.

Comparative Results and **Average** Optimization Impact: Table 13 compares the performance improvements of the GRA and RMCO methods over the results of the standard method for the six performance evaluation criteria. The GRA method provided an overall performance improvement (MSD) of 20.547%. The spot diagram showed improvements of 22.298% and 77.780% for Zoom 1 and Zoom 2, respectively. The distortion Zoom 1 and MTF Zoom 2 performances were also improved by 17.980% and 9.283%, respectively. The distortion Zoom 2 and MTF Zoom 1 showed slight performance losses of 2.823% and 1.235%, respectively. However, these losses were negligible compared to the other performance improvements, and hence, the overall performance improvement obtained by the GRA method remained relatively high (MSD = 20.547%). For the RMCO method, the overall performance improved by 55.182% compared to that of the standard process. Furthermore, with the exception of the MTF Zoom 2 objective, the RMCO method consistently outperformed the GRA method. 

Comparison of the differences between RMCO and GRA methods: The key advantage of RMCO over GRA lies in its closed-loop framework of “identification–constraint–iteration.” GRA begins by normalizing each objective’s signal-to-noise ratio (SNR), calculating the gray relational grade, and then assigning equal weights to all objectives to determine the parameters in a single step. This approach implicitly assumes equal importance and near-linear relationships among objectives, which can dilute critical indicators, resulting in a struggle to handle conflicting objectives. In contrast, RMCO leverages variance analysis to identify dominant factors and restricts or converges their levels based on fundamental decision rules. For conflicting objectives, it iteratively refines the search space through interval narrowing strategies. Built upon the initial GRA outcomes, RMCO incorporates heuristic tuning of secondary factors and conducts Pareto optimization across iterative experiments. This enables direct extraction of Pareto optimal solutions, offering better capture of nonlinear interactions and interdependencies, thereby enhancing overall robustness. Although this comes at the cost of an increased number of experiments, it yields a more consistent global trade-off optimum. In this study, RMCO achieved an average improvement of 55.182% in mean square deviation (MSD), significantly outperforming the 20.547% improvement attained by GRA. However, this gain required 24 additional experimental runs. Furthermore, for individual performance metrics such as MTF at Zoom 2, GRA occasionally delivered slightly better results, suggesting that RMCO prioritizes global trade-off optimality over localized peak performance.

### 5.4. Error Analysis

Owing to differences in computational simulation approaches across various software platforms, the models and data used in the MFA and CODE V(2023.03)analyses cannot be directly shared. Furthermore, the transition from discrete points to continuous functions introduces information loss during the conversion process. Ideally, if the analysis programs perfectly adhered to physical principles, denser data points would better maintain continuity and provide a more accurate model representation. In such cases, less data is lost during conversion, resulting in simulations that more closely match reality. Conversely, too few data points during conversion may lead to incomplete feature descriptions and exacerbate the inherent errors of finite element analysis (FEA). However, increasing the data density raises the computational costs and prolongs the analysis time. Thus, in this study, conversion errors were observed at three stages: the mesh size in MFA, refractive index regression, and fitting the deformed mesh to a smooth surface after MFA. All three errors arose from the transitions between discrete data and continuous functions. To address these issues, suitable conversion parameters were determined through testing, balancing the need to reduce the computational cost while simultaneously minimizing the conversion error.

Refractive Index Polynomial Regression: Polynomial regression of different orders produces different equations, each with a distinct fitting accuracy. However, the number of coefficients accepted by the CODE V(2023.03) software is limited. In particular, the user-defined gradient refractive indices are restricted to a maximum of 150 coefficients. A full three-variable eighth-order polynomial requires 165 coefficients, which exceeds this limit. Therefore, the present study only considered the first- through seventh-order polynomial fits. The Mean Absolute Error (MAE) was used as a quick and effective metric to evaluate the fitting accuracy in each case. Since the data range for evaluation matched the reference data range, generalization was not an issue. The analysis showed that the higher-order polynomials provided better fits. Consequently, all the refractive index regression analyses performed in this study used seventh-order polynomials.

Deformed Mesh-to-Surface Fitting Convergence: The primary experimental model used a mesh size of 0.025 mm, with the convergence analysis focusing on the hardness parameter. The fitting convergence study analyzed hardness values from 10,000 down to 0.0001. Using the “Point Deviation” command within the same software Rhinoceros 5.0, the numerical differences at the sample points were examined. The deformed mesh on the output surface of the standard process was chosen as the reference model, since its complex curvature provided a robust test. In other words, it was assumed that if an accurate fitting could be achieved for this complex surface, the same parameters would also be valid for the simpler input surface. The results, shown in Table 2, showed that as the hardness decreased, the node spacing differences also diminished. Both the maximum displacement and the average displacement converged at a hardness of 0.1. Excessively low hardness values can lead to overfitting beyond the sampled points. Thus, the convergence analysis determined that a hardness of 0.1 adequately captured the deformation characteristics caused by the injection molding process.

## 6. Conclusions

This study successfully combined mold flow analysis and optical design simulations to optimize the design and manufacturing of Alvarez freeform lenses produced by injection molding. Gray Relational Analysis (GRA) and Robust Multi-Criteria Optimization (RMCO) were employed to facilitate the multi-objective optimization of the key injection molding process parameters, achieving significant improvements in the optical performance compared with that obtained using single-objective Taguchi trials. The analyses revealed that the lens material, mold temperature, filling time, and pressure exhibited the most significant impact on the molding quality of the Alvarez lenses, as evaluated by the spot diagram, distortion, and modulation transfer function (MTF) performance measures across two zoom modes (wide-angle and telephoto). Of the two multi-objective optimization methods, RMCO required greater computational effort but showed a more substantial enhancement in the optical precision and stability of the manufactured lens.

Overall, the results presented in this study demonstrate the effectiveness of using advanced simulation and optimization techniques to improve the manufacturing process of precision optical components. The findings provide valuable insights for industry, highlighting the importance of controlling the process parameters to achieve high-quality products such as Alverez lenses. Future research is as follows: (1). To enable the transformation from discrete mesh data to a continuous refractive index field, a multivariate regression approach was adopted in this study. This method is particularly effective for optical components with smooth geometries, such as the Alvarez lens. However, for components exhibiting complex geometric discontinuities—such as Fresnel lenses and light guide plates—conventional regression fitting becomes insufficient. In such cases, neural networks or other deep learning-based methods are recommended to achieve more accurate modeling of the refractive index distribution. (2). In addition to manufacturing-induced variations in optical performance, the internal stress generated during the assembly of plastic lenses must also be considered, as it can introduce further degradation in optical characteristics. To address this issue, future work will incorporate finite element analysis (FEA) to simulate assembly-induced stress fields. Furthermore, inverse analysis will be employed to evaluate the impact of manufacturing parameters on the robustness of the combined manufacturing–assembly process.

## Figures and Tables

**Table 1:** Material properties of PMMA materials (ACRYREX CM205/CM211, CHIMEI).

Category	Data (CM 205)	Data (CM 211)	[Unit]
*ρ*: density	1.18	1.19	[g/cm^3^]
*µ*: viscosity	61.74–12.97 × 10^4^ (*T*: 210–250 °C; $\dot{\gamma }$: 10–10^5^)	87.25–25.84 × 10^4^ (*T*: 180–220 °C; $\dot{\gamma }$: 10–10^5^)	[g/(cm s)]
*ν*: Poisson ratio	0.38	0.38	[-]
*E*: Young’s modulus	2.8 × 10^10^	2.8 × 10^10^	[dyne/cm^2^]
*G*: torsion modulus	8.4 × 10^6^	8.4 × 10^6^	[dyne/cm^2^]
*α*: coefficient of thermal expansion	8 × 10^−5^	8 × 10^−5^	[1/K]
*k*: coefficient of heat conduction (*T*: 0–300 °C)	2.43 × 10^4^–2.53 × 10^4^	1.95 × 10^4^–3.06 × 10^4^	[erg/(s cm °C)]
*C_p_*: specific heat (*T*: 0–300 °C)	1.57 × 10^7^–2.75 × 10^7^	1.40 × 10^7^–2.20 × 10^7^	[erg/(g °C)]
*C_v_*: specific volume (*T*: 10–300 °C; *P*: 0–200 MPa)	8.01 × 10^−3^–9.55 × 10^−3^	8.08 × 10^−3^–9.62 × 10^−3^	[cm^3^/g]
Suggested mold temp.	50–70	30–40	[°C]
Suggested melt temp.	210–250	180–220	[°C]

**Table 2:** Convergence analysis of fitting surface (gray shadow presents the converged value).

Hardness	Maximum Distance	Average Distance
10,000	0.1	0.04209
1000	0.09993	0.02278
100	0.01711	0.001
10	0.00168	0.00005
1	0.00019	0.00001
0.1	0.00009	0.00001
0.01	0.00009	0.00001
0.001	0.00009	0.00001
0.0001	0.00009	0.00001

**Table 3:** Alvarez lens system design for wide-angle (Zoom 1) and telephoto (Zoom 2) modes.

		Wide-Angle (Zoom 1)	Telephoto (Zoom 2)
Field of View		50°	20°
Expected Zoom Factor		2.0 X	
Effective Focal Length		1.9863 mm	4.0229 mm
1st Set of Alvarez Lenses	1st Lens Shift (X direction)	−1.4172 mm	0.1912 mm
	2nd Lens Shift (X direction)	1.4172 mm	−0.1912 mm
2nd Set of Alvarez Lenses	1st Lens Shift (X direction)	0.7958 mm	−0.1521 mm
	2nd Lens Shift (X direction)	−0.7958 mm	0.1521 mm

**Table 4:** Field of view (FoV) data in optical simulations (Zoom 1 and Zoom 2).

	Field of View Information		
FOV No.	X-Angle	Y-Angle	Weighting
1	(0, 0)	(0, 0)	(1, 1)
2	(0, 0)	(12, 5)	(1, 1)
3	(0, 0)	(−12, −5)	(1, 1)
4	(0, 0)	(−25, −10)	(1, 1)
5	(0, 0)	(25, 10)	(1, 1)
6	(12, 5)	(0, 0)	(1, 1)
7	(−12, −5)	(0, 0)	(1, 1)
8	(25, 10)	(0, 0)	(1, 1)
9	(−25, −10)	(0, 0)	(1, 1)

**Table 5:** Factor level settings in orthogonal array for factor screening test.

	A	B	C	D	E	F	G	H	I	J	K
	Material	Mold Temp.	Melt Temp.	Filling Time	Flow Rate	Packing Time	Packing Pressure	V/P Switch	Eject Temp.	Cooling Time	-
Level 1	CM205	Min	Min	0.1 s	1	1 s	10%	95%	90 °C	10 s	-
Level 2	CM211	Max	Max	0.5 s	5	3 s	90%	99%	110 °C	20 s	-

**Table 6:** Factor screening experimental design (no shadow) and SNR results (gray shadows) for different quality outcomes.

Trials	A	B	C	D	E	F	G	H	I	J	Spot Diagram (SNR)		Distortion (SNR)		MTF (SNR)	
											Zoom 1	Zoom 2	Zoom 1	Zoom 2	Zoom 1	Zoom 2
1	CM205	Min	Min	0.1 s	1	1 s	0.1	0.95	90 °C	10 s	73.683	66.648	55.130	58.008	33.048	30.756
2	CM205	Min	Min	0.1 s	1	3 s	0.9	0.99	110 °C	20 s	70.291	62.301	55.908	55.644	35.258	31.768
3	CM205	Min	Max	0.5 s	5	1 s	0.1	0.95	110 °C	20 s	64.146	60.016	58.787	59.983	32.463	30.093
4	CM205	Max	Min	0.5 s	5	1 s	0.9	0.99	90 °C	10 s	68.389	59.037	59.221	54.356	39.896	25.836
5	CM205	Max	Max	0.1 s	5	3 s	0.1	0.99	90 °C	20 s	66.501	66.093	61.293	60.349	38.961	34.904
6	CM205	Max	Max	0.5 s	1	3 s	0.9	0.95	110 °C	10 s	71.801	58.223	62.362	49.992	38.166	23.924
7	CM211	Min	Max	0.5 s	1	1 s	0.9	0.99	90 °C	20 s	64.294	60.504	58.819	50.253	34.495	27.412
8	CM211	Min	Max	0.1 s	5	3 s	0.9	0.95	90 °C	10 s	69.944	67.312	62.871	59.224	36.312	31.594
9	CM211	Min	Min	0.5 s	5	3 s	0.1	0.99	110 °C	10 s	43.692	67.822	63.650	62.601	33.884	31.000
10	CM211	Max	Max	0.1 s	1	1 s	0.1	0.99	110 °C	10 s	64.369	62.161	59.234	56.309	32.958	32.314
11	CM211	Max	Min	0.5 s	1	3 s	0.1	0.95	90 °C	20 s	43.532	64.050	61.565	63.906	33.992	33.436
12	CM211	Max	Min	0.1 s	5	1 s	0.9	0.95	110 °C	20 s	65.865	60.927	60.032	56.275	37.954	29.596

**Table 7:** Factor screening results for significant factors for each quality measure.

Objectives		Significant Factor (L_12_ Factor Label)
Spot Diagram	Zoom 1	A, D/G
	Zoom 2	G, F
Distortion	Zoom 1	F, A
	Zoom 2	G, E
MTF	Zoom 1	B, G
	Zoom 2	G, D

**Table 8:** Factor levels settings for main test orthogonal array (gray shadows indicate fixed factors).

	A	B	C	D	E	F	Fixed	Fixed	Fixed	Fixed
	Material	Mold Temp.	Filling Time	Flow Rate	Packing Time	Packing Pressure	Melt Temp.	V/P Switch	Eject Temp.	Cooling Time
Level 1	CM 205	Min	0.1 s	1	1 s	10%	Max	97%	90 °C	10 s
Level 2	CM 211	25%	0.2 s	2	1.5 s	30%	Max	97%	90 °C	10 s
Level 3		50%	0.3 s	3	2 s	50%	Max	97%	90 °C	10 s
Level 4		75%	0.4 s	4	2.5 s	70%	Max	97%	90 °C	10 s
Level 5		Max	0.5 s	5	3 s	90%	Max	97%	90 °C	10 s
STD	CM 205	50%	0.2 s	3	3 s	50%	50%	97%	100 °C	15 s

**Table 9:** SNR results obtained for different quality outcomes using the main test orthogonal array (gray shadow indicates the orthogonal array).

Trials	A	B	C	D	E	F	Spot Diagram (SNR)		Distortion (SNR)		MTF (SNR)	
							Zoom 1	Zoom 2	Zoom 1	Zoom 2	Zoom 1	Zoom 2
1	CM 205	Min	0.1 s	1	1 s	10%	63.408	61.451	57.915	58.022	31.059	30.120
2	CM 205	25%	0.2 s	2	1.5 s	30%	63.459	62.168	58.003	58.541	30.832	29.391
3	CM 205	50%	0.3 s	3	2 s	50%	63.614	67.319	57.980	62.802	29.030	25.226
4	CM 205	75%	0.4 s	4	2.5 s	70%	68.493	65.055	65.732	59.710	32.482	30.348
5	CM 205	Max	0.5 s	5	3 s	90%	65.402	62.102	61.598	53.803	32.581	24.889
6	CM 211	Min	0.2 s	3	2.5 s	90%	68.499	70.161	62.146	58.377	33.072	27.686
7	CM 211	25%	0.3 s	4	3 s	10%	64.335	63.148	59.722	60.548	31.547	28.699
8	CM 211	50%	0.4 s	5	1 s	30%	64.789	61.137	59.741	58.557	32.148	29.104
9	CM 211	75%	0.5 s	1	1.5 s	50%	64.862	59.996	61.005	53.867	29.067	28.877
10	CM 211	Max	0.1 s	2	2 s	70%	65.019	66.668	59.570	66.568	22.014	29.021
11	CM 205	Min	0.3 s	5	1.5 s	70%	71.583	68.791	65.341	58.371	33.689	27.065
12	CM 205	25%	0.4 s	1	2 s	90%	64.037	54.883	58.348	54.155	32.872	20.428
13	CM 205	50%	0.5 s	2	2.5 s	10%	63.157	59.213	57.710	56.045	30.600	25.704
14	CM 205	75%	0.1 s	3	3 s	30%	62.387	62.567	56.898	62.479	30.277	30.493
15	CM 205	Max	0.2 s	4	1 s	50%	64.634	64.733	59.372	62.303	30.455	29.913
16	CM 211	Min	0.4 s	2	3 s	50%	64.878	59.424	60.253	57.899	31.207	24.564
17	CM 211	25%	0.5 s	3	1 s	70%	67.897	57.225	59.611	46.466	30.626	23.552
18	CM 211	50%	0.1 s	4	1.5 s	90%	69.031	70.489	61.585	60.658	36.969	28.528
19	CM 211	75%	0.2 s	5	2 s	10%	63.828	64.227	58.953	64.486	31.875	32.311
20	CM 211	Max	0.3 s	1	2.5 s	30%	63.631	64.279	59.539	59.256	30.550	28.104
21	CM 205	Min	0.5 s	4	2 s	30%	65.529	58.640	58.651	54.487	23.870	24.667
22	CM 205	25%	0.1 s	5	2.5 s	50%	65.302	63.070	59.161	60.652	31.036	22.467
23	CM 205	50%	0.2 s	1	3 s	70%	72.077	67.540	64.826	61.756	32.836	37.333
24	CM 205	75%	0.3 s	2	1 s	90%	63.024	58.397	57.500	57.507	35.859	18.652
25	CM 205	Max	0.4 s	3	1.5 s	10%	63.899	64.469	58.650	62.741	31.793	29.308
STD							64.316	60.241	58.947	61.314	29.563	29.440

**Table 10:** Gray relational coefficient and gray relational grade for all quality measures (gray shadow indicates gray relational grades).

Trials	Spot Diagram		Distortion		MTF		*Γ_i_*
	Zoom 1	Zoom 2	Zoom 1	Zoom 2	Zoom 1	Zoom 2	
	*γ_i,_*_1_	*γ_i,_*_2_	*γ_i,_*_3_	*γ_i,_*_4_	*γ_i,_*_5_	*γ_i,_*_6_	
1	0.359	0.463	0.361	0.540	0.559	0.564	0.474
2	0.360	0.484	0.364	0.556	0.549	0.540	0.476
3	0.364	0.711	0.363	0.727	0.485	0.436	0.514
4	0.575	0.590	1.000	0.594	0.625	0.572	0.659
5	0.421	0.482	0.517	0.441	0.630	0.429	0.486
6	0.575	0.960	0.552	0.551	0.657	0.492	0.631
7	0.385	0.515	0.424	0.625	0.580	0.520	0.508
8	0.399	0.455	0.424	0.556	0.608	0.532	0.496
9	0.402	0.426	0.483	0.442	0.486	0.525	0.461
10	0.407	0.671	0.418	1.000	0.333	0.529	0.560
11	0.907	0.821	0.919	0.551	0.695	0.476	0.728
12	0.376	0.333	0.374	0.447	0.646	0.356	0.422
13	0.352	0.409	0.355	0.489	0.540	0.445	0.432
14	0.333	0.496	0.333	0.711	0.528	0.577	0.496
15	0.394	0.575	0.410	0.702	0.534	0.557	0.529
16	0.402	0.414	0.446	0.537	0.565	0.422	0.464
17	0.537	0.370	0.419	0.333	0.541	0.404	0.434
18	0.614	1.000	0.516	0.630	1.000	0.515	0.712
19	0.370	0.555	0.395	0.828	0.595	0.650	0.565
20	0.365	0.557	0.416	0.579	0.538	0.503	0.493
21	0.425	0.397	0.384	0.454	0.363	0.424	0.408
22	0.417	0.513	0.402	0.629	0.558	0.386	0.484
23	1.000	0.726	0.830	0.676	0.644	1.000	0.813
24	0.349	0.392	0.349	0.526	0.871	0.333	0.470
25	0.372	0.564	0.384	0.724	0.591	0.538	0.529

**Table 11:** Factor level settings for iterative tests: (**a**) GRA and (**b**) RMCO (gray shadows indicate fixed factors).

**(a)**	**Material**	**Mold** **Temperature**	**Filling** **Time**	**Flow** **Rate**	**Packing** **Time**	**Packing** **Pressure**
Level 1	CM 205	Min	0.1 s	1	1 s	10%
Level 2	CM 211	25%	0.2 s	2	1.5 s	30%
Level 3		50%	0.3 s	3	2 s	50%
Level 4		75%	0.4 s	4	2.5 s	70%
Level 5		Max	0.5 s	5	3 s	90%

**(b)**	**Material**	**Mold** **Temperature**	**Filling** **Time**	**Flow** **Rate**	**Packing** **Time**	**Packing** **Pressure**
Level 1	CM 211	Min	0.1 s	4	1.5 s	70%
Level 2	CM 211	25%	0.125 s	4	1.5 s	75%
Level 3	CM 211	50%	0.15 s	4	1.5 s	80%
Level 4	CM 211	75%	0.175 s	4	1.5 s	85%
Level 5	CM 211	Max	0.2 s	4	1.5 s	90%

**Table 12:** MSD improvements obtained in RMCO iterative testing (gray shadow indicates the greatest improvement).

Trials	A	B	C	D	E	F	Spot Diagram				Distortion				MTF				Average Improve
							Zoom 1		Zoom 2		Zoom 1		Zoom 2		Zoom 1		Zoom 2		
							MSD	Increase	MSD	Increase	MSD	Increase	MSD	Increase	MSD	Increase	MSD	Increase	
1	CM 211	Min	0.1 s	4	1.5 s	70%	2.15 × 10^−7^	41.84%	2.18 × 10^−7^	76.92%	9.24 × 10^−7^	27.53%	6.54 × 10^−7^	11.44%	7.59 × 10^−4^	31.41%	1.35 × 10^−3^	−18.81%	28.388%
2	CM 211	25%	0.125 s	4	1.5 s	75%	1.76 × 10^−7^	52.36%	1.91 × 10^−7^	79.76%	8.49 × 10^−7^	33.35%	4.78 × 10^−7^	35.37%	8.81 × 10^−4^	20.34%	1.05 × 10^−3^	7.41%	38.100%
3	CM 211	50%	0.15 s	4	1.5 s	80%	1.55 × 10^−7^	58.16%	2.21 × 10^−7^	76.66%	6.39 × 10^−7^	49.86%	6.79 × 10^−7^	8.16%	7.85 × 10^−4^	29.00%	1.03 × 10^−3^	9.09%	38.490%
4	CM 211	75%	0.175 s	4	1.5 s	85%	7.85 × 10^−8^	78.80%	3.28 × 10^−7^	65.32%	4.12 × 10^−7^	67.66%	1.09 × 10^−6^	−48.16%	4.15 × 10^−4^	62.49%	1.20 × 10^−3^	−5.42%	36.782%
5	CM 211	Max	0.2 s	4	1.5 s	90%	9.48 × 10^−8^	74.40%	2.87 × 10^−7^	69.70%	4.25 × 10^−7^	66.63%	6.88 × 10^−7^	6.94%	2.96 × 10^−4^	73.27%	1.01 × 10^−3^	11.59%	50.423%
6	CM 211	Min	0.125 s	4	1.5 s	90%	1.46 × 10^−7^	60.68%	3.47 × 10^−7^	63.27%	5.01 × 10^−7^	60.69%	1.53 × 10^−6^	−107.54%	3.07 × 10^−4^	72.21%	1.10 × 10^−3^	2.94%	25.375%
7	CM 211	25%	0.15 s	4	1.5 s	70%	2.07 × 10^−7^	44.19%	2.18 × 10^−7^	76.92%	8.35 × 10^−7^	34.52%	7.31 × 10^−7^	1.08%	7.43 × 10^−4^	32.81%	9.69 × 10^−4^	14.80%	34.052%
8	CM 211	50%	0.175 s	4	1.5 s	75%	2.07 × 10^−7^	43.95%	2.41 × 10^−7^	74.52%	7.73 × 10^−7^	39.36%	7.29 × 10^−7^	1.34%	8.86 × 10^−4^	19.83%	8.47 × 10^−4^	25.56%	34.096%
9	CM 211	75%	0.2 s	4	1.5 s	80%	1.62 × 10^−7^	56.30%	2.53 × 10^−7^	73.25%	5.18 × 10^−7^	59.39%	8.77 × 10^−7^	−18.71%	8.33 × 10^−4^	24.67%	8.23 × 10^−4^	27.70%	37.100%
**10**	**CM 211**	**Max**	**0.1 s**	**4**	**1.5 s**	**85%**	**1.13 × 10^−7^**	**69.58%**	**5.82 × 10^−8^**	**93.85%**	**4.56 × 10^−7^**	**64.19%**	**5.18 × 10^−7^**	**29.88%**	**3.78 × 10^−4^**	**65.84%**	**1.05 × 10^−3^**	**7.75%**	**55.182%**
11	CM 211	Min	0.15 s	4	1.5 s	85%	1.01 × 10^−7^	72.69%	1.10 × 10^−7^	88.33%	4.46 × 10^−7^	65.03%	9.21 × 10^−7^	−24.58%	7.57 × 10^−4^	31.51%	9.49 × 10^−4^	16.62%	41.601%
12	CM 211	25%	0.175 s	4	1.5 s	90%	6.29 × 10^−8^	83.02%	1.87 × 10^−7^	80.22%	6.51 × 10^−7^	48.93%	1.36 × 10^−6^	−83.64%	5.70 × 10^−4^	48.44%	8.05 × 10^−4^	29.21%	34.364%
13	CM 211	50%	0.2 s	4	1.5 s	70%	2.33 × 10^−7^	37.17%	1.79 × 10^−7^	81.03%	8.69 × 10^−7^	31.82%	7.33 × 10^−7^	0.83%	8.04 × 10^−4^	27.29%	9.20 × 10^−4^	19.11%	32.874%
14	CM 211	75%	0.1 s	4	1.5 s	75%	2.18 × 10^−7^	41.07%	2.51 × 10^−7^	73.44%	9.23 × 10^−7^	27.62%	2.87 × 10^−7^	61.11%	9.26 × 10^−4^	16.29%	9.83 × 10^−4^	13.58%	38.849%
15	CM 211	Max	0.125 s	4	1.5 s	80%	1.25 × 10^−7^	66.11%	1.36 × 10^−7^	85.65%	5.82 × 10^−7^	54.33%	5.65 × 10^−7^	23.50%	5.75 × 10^−4^	47.98%	8.08 × 10^−4^	29.00%	51.097%
16	CM 211	Min	0.175 s	4	1.5 s	80%	1.59 × 10^−7^	57.10%	1.00 × 10^−7^	89.39%	7.53 × 10^−7^	40.89%	6.55 × 10^−7^	11.40%	8.30 × 10^−4^	24.90%	1.40 × 10^−3^	−22.69%	33.498%
17	CM 211	25%	0.2 s	4	1.5 s	85%	5.94 × 10^−8^	83.95%	1.32 × 10^−7^	86.03%	6.01 × 10^−7^	52.88%	9.41 × 10^−7^	−27.31%	5.22 × 10^−4^	52.82%	1.22 × 10^−3^	−7.20%	40.197%
18	CM 211	50%	0.1 s	4	1.5 s	90%	1.25 × 10^−7^	66.23%	8.93 × 10^−8^	90.55%	6.94 × 10^−7^	45.53%	8.59 × 10^−7^	−16.29%	2.01 × 10^−4^	81.83%	1.40 × 10^−3^	−23.36%	40.748%
19	CM 211	75%	0.125 s	4	1.5 s	70%	2.45 × 10^−7^	33.82%	2.72 × 10^−7^	71.19%	1.06 × 10^−6^	16.50%	5.48 × 10^−7^	25.89%	9.32 × 10^−4^	15.71%	1.44 × 10^−3^	−26.68%	22.738%
20	CM 211	Max	0.15 s	4	1.5 s	75%	2.05 × 10^−7^	44.72%	2.98 × 10^−7^	68.50%	1.03 × 10^−6^	19.54%	4.85 × 10^−7^	34.32%	9.09 × 10^−4^	17.77%	1.57 × 10^−3^	−37.66%	24.529%
21	CM 211	Min	0.2 s	4	1.5 s	75%	2.00 × 10^−7^	46.11%	1.08 × 10^−7^	88.58%	8.51 × 10^−7^	33.20%	1.18 × 10^−6^	−59.64%	8.62 × 10^−4^	22.04%	1.95 × 10^−3^	−71.02%	9.877%
22	CM 211	25%	0.1 s	4	1.5 s	80%	1.59 × 10^−7^	57.16%	2.75 × 10^−7^	70.92%	7.28 × 10^−7^	42.87%	3.73 × 10^−6^	−404.13%	6.79 × 10^−4^	38.63%	1.37 × 10^−3^	−20.45%	−35.832%
23	CM 211	50%	0.125 s	4	1.5 s	85%	9.70 × 10^−8^	73.80%	2.26 × 10^−7^	76.07%	5.06 × 10^−7^	60.28%	3.02 × 10^−6^	−308.28%	4.16 × 10^−4^	62.38%	9.89 × 10^−4^	13.11%	−3.772%
24	CM 211	75%	0.15 s	4	1.5 s	90%	1.15 × 10^−7^	68.86%	1.66 × 10^−7^	82.41%	4.91 × 10^−7^	61.50%	1.31 × 10^−6^	−77.95%	2.69 × 10^−4^	75.71%	8.34 × 10^−4^	26.66%	39.531%
25	CM 211	Max	0.175 s	4	1.5 s	70%	2.33 × 10^−7^	36.96%	3.51 × 10^−7^	62.91%	9.87 × 10^−7^	22.58%	3.74 × 10^−6^	−405.50%	9.60 × 10^−4^	13.19%	9.89 × 10^−4^	13.05%	−42.802%
**STD**							3.70 × 10^−7^	-	9.46 × 10^−7^	-	1.27 × 10^−6^	-	7.39 × 10^−7^	0.00%	1.11 × 10^−3^	-	1.14 × 10^−3^	-	-

**Table 13:** Performance improvement comparison of STD, GRA, and RMCO.

		Spot Diagram		Distortion		MTF		Average Improvement
		Zoom 1	Zoom 2	Zoom 1	Zoom 2	Zoom 1	Zoom 2	
STD	MSD	3.702 × 10^−7^	9.459 × 10^−7^	1.275 × 10^−6^	7.390 × 10^−7^	1.106 × 10^−3^	1.138 × 10^−3^	-
GRA	MSD	2.876 × 10^−7^	2.102 × 10^−7^	1.045 × 10^−6^	7.598 × 10^−7^	1.119 × 10^−3^	1.032 × 10^−3^	20.547%
	Increase Ratio	22.298%	77.780%	17.980%	−2.823%	−1.235%	9.283%	
RMCO	MSD	1.126 × 10^−7^	5.818 × 10^−8^	4.564 × 10^−7^	5.181 × 10^−7^	3.777 × 10^−4^	1.050 × 10^−3^	55.182%
	Increase Ratio	69.583%	93.849%	64.194%	29.881%	65.842%	7.747%	

## Data Availability

The original contributions presented in this study are included in the article. Further inquiries can be directed to the corresponding author.
